# Diffusion tensor imaging along the perivascular space: the bias from crossing fibres

**DOI:** 10.1093/braincomms/fcae421

**Published:** 2024-11-21

**Authors:** Charalampos Georgiopoulos, Alice Werlin, Samo Lasic, Sara Hall, Danielle van Westen, Nicola Spotorno, Oskar Hansson, Markus Nilsson

**Affiliations:** Department of Clinical Sciences, Diagnostic Radiology, Medical Faculty, Lund University, 221 85 Lund, Sweden; Image and Function, Skåne University Hospital, 221 85 Lund, Sweden; Department of Clinical Sciences, Diagnostic Radiology, Medical Faculty, Lund University, 221 85 Lund, Sweden; Department of Clinical Sciences, Diagnostic Radiology, Medical Faculty, Lund University, 221 85 Lund, Sweden; Department of Clinical Sciences Malmö, Clinical Memory Research Unit, Faculty of Medicine, Lund University, 211 46 Malmö, Sweden; Memory Clinic, Skåne University Hospital, 205 02 Malmö, Sweden; Department of Clinical Sciences, Diagnostic Radiology, Medical Faculty, Lund University, 221 85 Lund, Sweden; Image and Function, Skåne University Hospital, 221 85 Lund, Sweden; Department of Clinical Sciences Malmö, Clinical Memory Research Unit, Faculty of Medicine, Lund University, 211 46 Malmö, Sweden; Department of Clinical Sciences Malmö, Clinical Memory Research Unit, Faculty of Medicine, Lund University, 211 46 Malmö, Sweden; Memory Clinic, Skåne University Hospital, 205 02 Malmö, Sweden; Department of Clinical Sciences, Diagnostic Radiology, Medical Faculty, Lund University, 221 85 Lund, Sweden

**Keywords:** Parkinson’s disease, progressive supranuclear palsy, glymphatic system, diffusion, MRI

## Abstract

Non-invasive evaluation of glymphatic function has emerged as a crucial goal in neuroimaging, and diffusion tensor imaging along the perivascular space (DTI-ALPS) has emerged as a candidate method for this purpose. Reduced ALPS index has been suggested to indicate impaired glymphatic function. However, the potential impact of crossing fibres on the ALPS index has not been assessed, which was the aim of this cross-sectional study. For this purpose, we used DTI-ALPS in a cohort with three groups: Parkinson’s disease (PD) (*n* = 60, mean age 63.3 ± 1.5, 33 males), progressive supranuclear palsy (PSP) (*n* = 17, mean age 70.9 ± 1.5, 9 males) and healthy controls (*n* = 41, mean age 64.5 ± 8.4, 15 males). The ALPS index was calculated blinded to diagnosis, by manually placing two sets of regions of interest (ROI) on the projection and association fibres of each hemisphere. Annotation was performed twice: once on conventional diffusion-encoded colour maps weighted by fractional anisotropy and once on maps with weights adjusted for high incidence of crossing fibres. PSP patients had significantly lower conventional ALPS indices compared with both healthy controls (right hemisphere: *P* = 0.009; left hemisphere: *P* < 0.001) and PD patients (right hemisphere: *P* = 0.024; left hemisphere: *P* < 0.001). There were no differences between healthy controls and PD patients. After adjusting the ROI to avoid regions of crossing fibres, the ALPS index significantly decreased in healthy controls (right hemisphere: *P* < 0.001; left hemisphere: *P* < 0.001) and PD (right hemisphere: *P* < 0.001; left hemisphere: *P* < 0.001). In PSP, the adjusted ALPS index was lower compared with the conventional one only in the right hemisphere (*P* = 0.047). Overall, this adjustment led to less significant differences among diagnostic groups. Specifically, with the adjusted ALPS index, PSP patients showed significantly lower ALPS index compared with healthy controls (right hemisphere: *P* = 0.044; left hemisphere: *P* = 0.029) and PD patients (*P* = 0.003 for the left hemisphere only). Our results suggest that crossing fibres significantly inflate the ALPS index and should be considered a critical pitfall of this method. This factor could partly explain the variability observed in previous studies. Unlike previous research, we observed no differences between PD and healthy controls, likely because most patients in our cohort were in the early phase of the disease. Thus, the ALPS index may not be a sensitive indicator of glymphatic function at least in the initial stages of neurodegeneration in PD.

## Teaser

Georgiopoulos *et al*. report that crossing fibres significantly affect diffusion tensor imaging along the perivascular space, raising concerns about its reliability as a measure of glymphatic function. Additionally, they found no differences between healthy controls and early Parkinson’s disease patients, underscoring the method’s limitations at early stages of neurodegeneration.

## Graphical Abstract

**Graphical Abstract:**
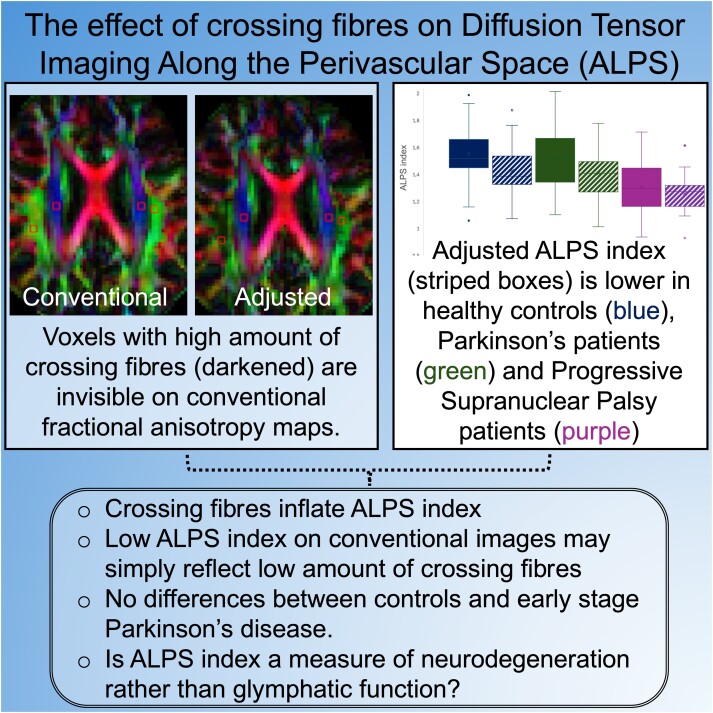

## Introduction

The central nervous system lacks a conventional lymphatic system, but in 2012, an alternative waste clearing system in the brain was described and named the glymphatic system.^1^ This breakthrough has led to a greater understanding of fluid dynamics in the brain and the way the brain rids itself from waste products.^2^ Additionally, glymphatic dysfunction has been linked to a range of neurodegenerative disorders, including Alzheimer’s and Parkinson’s disease (PD), highlighting its potential as a target for pharmacological intervention.^3,4^ As a result, the *in vivo* visualization and assessment of glymphatic flow in humans have emerged as a notable area of research.

Several imaging techniques have been proposed for mapping the glymphatic system, each offering its unique advantages and limitations. An invasive MRI method, where a contrast agent is injected intrathecally, is considered the ‘gold standard’ in the field.^5^ However, the method is invasive, associated with side effects and potential toxicity, and demands serial MRI imaging. In 2017, Taoka *et al*. introduced diffusion tensor imaging along the perivascular space (DTI-ALPS) as a non-invasive, indirect method to assess glymphatic activity by calculating the ALPS index as a tentative measure of water diffusion in the perivascular space.^6^ This method was developed in an effort to evaluate diffusion within the perivascular spaces of the medullary veins, a crucial component of the glymphatic system, by assessing the diffusion of free water in the interstitium while minimizing the strong influence of the white matter fibres that dominate in diffusion images.^7,8^ The use of DTI-ALPS in research has surged in the last 2 years, with PubMed indexing about 34 articles in 2022 and 64 in 2023.

PD is one of the most common neurodegenerative disorders.^9,10^ It is marked by the accumulation of alpha-synuclein and the loss of dopaminergic neurons in the substantia nigra, leading to symptoms such as tremor, rigidity, postural instability and bradykinesia.^11,12^ Impairment of the glymphatic system and neuroinflammation are believed to worsen disease progression.^13,14^ Progressive supranuclear palsy (PSP) is a rarer condition that mirrors PD in symptoms but is characterized by rapid progression and distinct features such as early postural instability, vertical gaze palsy and accumulation of hyperphosphorylated tau protein isoforms.^15^ DTI-ALPS has been applied in several studies involving patients with PD, but, to our knowledge, has not been used on patients with PSP.^16-24^ To better understand the current state of research on DTI-ALPS in PD, we reviewed the existing literature, aiming to highlight the variability in methodologies and findings across studies. Table 1 summarizes the existing literature on the use of DTI-ALPS in PD, focusing on technical details (acquisition parameters and ROI placement), demographics and reported ALPS values.

**Table 1:** Overview of the application of DTI-ALPS in studying PD—a literature review summary

Study	TR/TE	*b*-value	Encoding directions	ROI placement	Participants	Number	Age (y)	Sex (M/F)	UPDRS	ALPS index	Effect size (Cohen’s *d*) for ALPS
Si *et al*.^16^	8000/80 ms	1000 s/mm^2^	30	Left hemisphere	Healthy controls	129	61.96 ± 7.21	59/70		1.31 ± 0.17	0.65
					PD patients	168	59.85 ± 9.88	96/72	21.64 ± 12.37	1.20 ± 0.17	
Bae *et al*.^17^	9900/77 ms	1000 s/mm^2^	32	Both hemispheres	Healthy controls	54	69.0 ± 10.5	23/31		1.66 ± 0.20	0.71
					PD patients	54	68.9 ± 9.4	23/31	16.6 ± 7.0	1.51 ± 0.22	
Cai *et al*.^18^	5100/130 ms	1000 s/mm^2^	25	Both hemispheres	Healthy controls	42	61.52 ± 7.54	19/23		1.31 ± 0.18	0.83
					PD patients	93	61.87 ± 8.52	49/44	35.04 ± 12.52	1.16 ± 0.18	
Shen *et al*.^20^	6000/71.8 ms	1000 s/mm^2^	not	Both hemispheres	Early healthy controls	47	52.22 ± 8.84	19/18		1.52 ± 0.22	0.52
		and	reported		Early PD patients	40	54.55 ± 8.34	21/19	19.05 ± 11.28	1.39 ± 0.28	
		3000 s/mm^2^			Late healthy controls	31	57.86 ± 5.67	11/20		1.57 ± 0.19	0.9
					Late PD patients	36	59.72 ± 10.13	16/20	33.14 ± 8.83	1.36 ± 0.27	
Ma *et al*.^21^	5472/93 ms	1000 s/mm^2^	31	Left hemisphere	Healthy controls	36	62 ± 6.24	18/18		1.53 ± 0.16	0.45 (controls versus early PD)
					Early PD patients	35	63.57 ± 8.93	17/18	26.31 ± 9.8	1.46 ± 0.15	0.65 (controls versus late PD)
					Late PD patients	36	65.75 ± 7.23	14/22	35.39 ± 0.54	1.44 ± 0.16	
McKnight	10 000/60 ms	1000 s/mm^2^	33	Both hemispheres	Essential Tremor	37	67.8 ± 6.2	21/16		1.48 ± 0.16	0.19
*et al.* ^22^					PD patients	144	62.2 ± 9	97/47	9.2 ± 4.5	1.45 ± 0.16	
Gu *et al*.^23^	8000/80 ms	not	not	Left hemisphere	Healthy controls	106	60.3 ± 7	40/66		1.34 ± 0.19	0.68
		reported	reported		PD patients	124	60.7 ± 7.25	44/80	22.82 ± 12.3	1.22 ± 0.18	
Qin *et al*.^24^	900/88 ms	1000 s/mm^2^	64	Both hemispheres	Healthy controls	67	60.10 ± 10.562	43/24		1.55 ± 0.243	0.37
					PD patients	153	60.97 ± 9.47	99/54	20.76 ± 9.03	1.46 ± 0.243	

Values for age, UPDRS and ALPS index are presented in the form om mean ± standard deviation.

TR, repetition time; TE, echo time; y, years; M, male; F, female; UPDRS, Unified Parkinson’s Disease Rating Scale; ALPS, along the perivascular space; PD, Parkinson’s disease.

**Figure 1:**
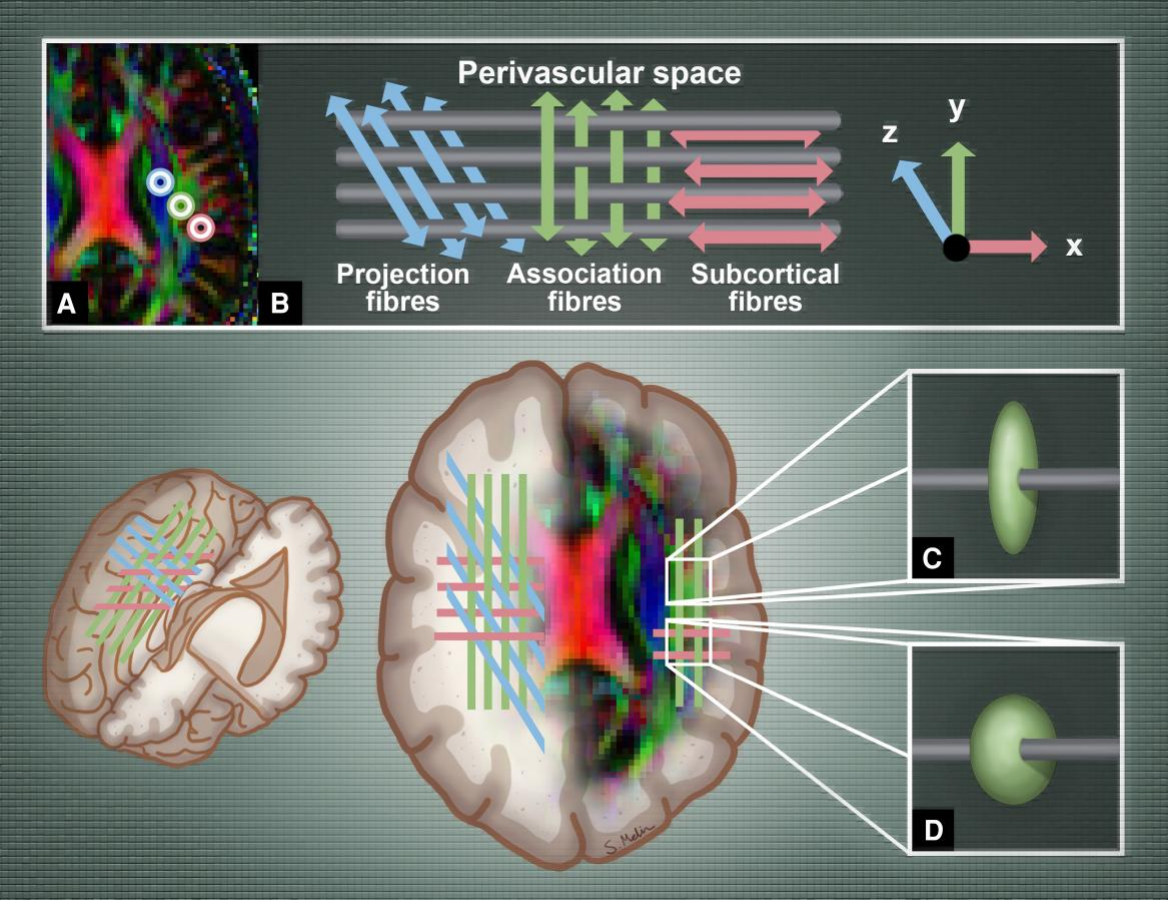
**Schematic representation of the main fibres at the level of the lateral ventricular body, illustrating how crossing fibres affect the diffusion tensor. A**. Placement of ROI as per Taoka *et al*.^7^  **B**. Projection fibres (blue) are oriented along the *z*-axis, association fibres (green) along the *y*-axis and subcortical fibres along the *x*-axis. The perivascular space of the medullary veins is also oriented along the *x*-axis. **C**. Example of an ROI within association fibres, showing no crossing fibres. **D**. Example of an ROI within association fibres, where crossing fibres intersect perpendicularly. Notice how the ellipsoid changes shape but not colour, since the main diffusion component is still along the *y*-axis.

Although the ALPS index shows a strong correlation with glymphatic function as measured by contrast-enhanced MRI,^25^ DTI-ALPS is sensitive to various factors, including head positioning and imaging parameters.^26^ Recently, its validity to assess the glymphatic system has come under scrutiny, as it measures diffusion in the brain’s deep white matter, whereas glymphatic activity is primarily observed near the brain’s surface.^27^ Furthermore, the method is based on diffusion tensor imaging (DTI), which describes the diffusion of water in three dimensions. This method is well suited for detecting tissue anisotropy in coherent bundles of fibres. However, it faces challenges in areas where fibres cross, as depicted in Fig. 1.^28^ A large portion of brain voxels hosts multiple fibre bundles oriented in various directions within each imaging voxel, meaning the diffusion tensor is a poor representation of tissue anisotropy in these regions. This issue, referred to as the ‘crossing fibre problem’, is estimated to affect up to 90% of the white matter and is a fundamental limitation of DTI,^29^ which can lead to potential misinterpretations.^30^ Surprisingly, the effect of crossing fibres on DTI-ALPS has not been previously addressed.

In this study, we aimed to examine the influence of crossing fibres on the ALPS index, hypothesizing that the ALPS index would decrease if areas with high levels of crossing fibres are excluded from the measurement. Additionally, we sought to replicate and extend previous research on PD by comparing the ALPS index in PD and PSP patients against healthy controls.

## Materials and methods

### Methodological background

The ALPS method was developed to measure the impact of diffusion of water molecules within the perivascular spaces along deep medullary veins, which are aligned in the *x*-direction (left–right) at the level of the lateral ventricular body. In this region, DTI is predominantly influenced by projection and association fibres that run perpendicular to the *x*-direction, specifically in the *z*-direction (superior–inferior) and *y*-direction (anterior–posterior), respectively. The ALPS index aims to be sensitive to the perivascular diffusion along the *x*-axis, and it is defined as


(1) $$\mathrm{ALPS}=\frac{D_{xx,\mathrm{proj}}+D_{xx,\mathrm{assoc}}}{D_{yy,\mathrm{proj}}+D_{zz,\mathrm{assoc}}}$$

where $D_{xx,\mathrm{proj}}$ and $D_{yy,\mathrm{proj}}$ are diffusion tensor components for the projection fibres and $D_{xx,\mathrm{assoc}}$ and $D_{zz,\mathrm{assoc}}$ for the association fibres.^6^ This equation assumes there are no crossing fibres, and would, in an ideal situation, be sensitive to perivascular flow.

For simplicity, let us assume that all fibres are axially symmetric with radial diffusivity, $D_{\mathrm{rad}}$ and the perivascular flow is reflected in diffusivity $D_{\mathrm{pv}}$ along *x*-axis, such that $D_{xx,\mathrm{proj}}=D_{xx,\mathrm{assoc}}=D_{\mathrm{rad}}+D_{\mathrm{pv}}$, $D_{yy,\mathrm{proj}}=D_{zz,\mathrm{assoc}}=D_{\mathrm{rad}}$. In such a situation, the true ALPS index is given by


(2) $$\mathrm{ALPS}=1+\frac{D_{\mathrm{pv}}}{D_{\mathrm{rad}}}$$

According to Equation (2), the ALPS index increases with perivascular diffusivity $D_{\mathrm{pv}}$ and this increase depends on the radial diffusivity of the fibres $D_{\mathrm{rad}}$. Less anisotropic fibres, with higher $D_{\mathrm{rad}}$, will reduce the ALPS index. Thus, fibre anisotropy determines the sensitivity of the ALPS index to the perivascular flow.

To analyse the potential impact of crossing fibres on the ALPS index, let us introduce volume fractions $f_{\mathrm{Xp}}$ and $f_{\mathrm{Xa}}$ of fibres that cross projection and association fibres, respectively, so that the resulting diffusivities are:


(3) $$\begin{array}{r} D_{xx,\mathrm{proj}+\mathrm{cross}}=(1-f_{\mathrm{Xp}})D_{xx,\mathrm{proj}}+f_{\mathrm{Xp}}D_{xx,\mathrm{cross}}\\ D_{yy,\mathrm{proj}+\mathrm{cross}}=(1-f_{\mathrm{Xp}})D_{yy,\mathrm{proj}}+f_{\mathrm{Xp}}D_{yy,\mathrm{cross}}\\ D_{xx,\mathrm{assoc}+\mathrm{cross}}=(1-f_{\mathrm{Xa}})D_{xx,\mathrm{assoc}}+f_{\mathrm{Xa}}D_{xx,\mathrm{cross}}\\ D_{zz,\mathrm{assoc}+\mathrm{cross}}=(1-f_{\mathrm{Xa}})D_{zz,\mathrm{assoc}}+f_{\mathrm{Xa}}D_{zz,\mathrm{cross}} \end{array}$$

The apparent ALPS index as measured in the presence of crossing fibres then becomes:


(4) $$\mathrm{ALPSX}=\frac{D_{xx,\mathrm{proj}+\mathrm{cross}}+D_{xx,\mathrm{assoc}+\mathrm{cross}}}{D_{yy,\mathrm{proj}+\mathrm{cross}}+D_{zz,\mathrm{assoc}+\mathrm{cross}}}.$$

Such crossing fibres could give rise to an apparent value of ALPS > 1 even in the absence of glymphatic system and diffusion in the perivascular space.

Consider fibres crossing only at one site, either in the projection or the association fibres, so that the volume fractions of crossing fibres are $f_{\mathrm{Xp}}=f_{X}$ and $f_{\mathrm{Xa}}=0$ or $f_{\mathrm{Xp}}=0$ and $f_{\mathrm{Xa}}=f_{X}$. In this case, the apparent ALPS index (ALPSX) from Equation (4) is given by:


(5) $$\mathrm{ALPSX}=\mathrm{ALPS}+\frac{\;f_{X}}{2}\left(\frac{D_{xx,\mathrm{cross}}}{D_{\mathrm{rad}}}-\mathrm{ALPS}\right)$$

**Figure 2:**
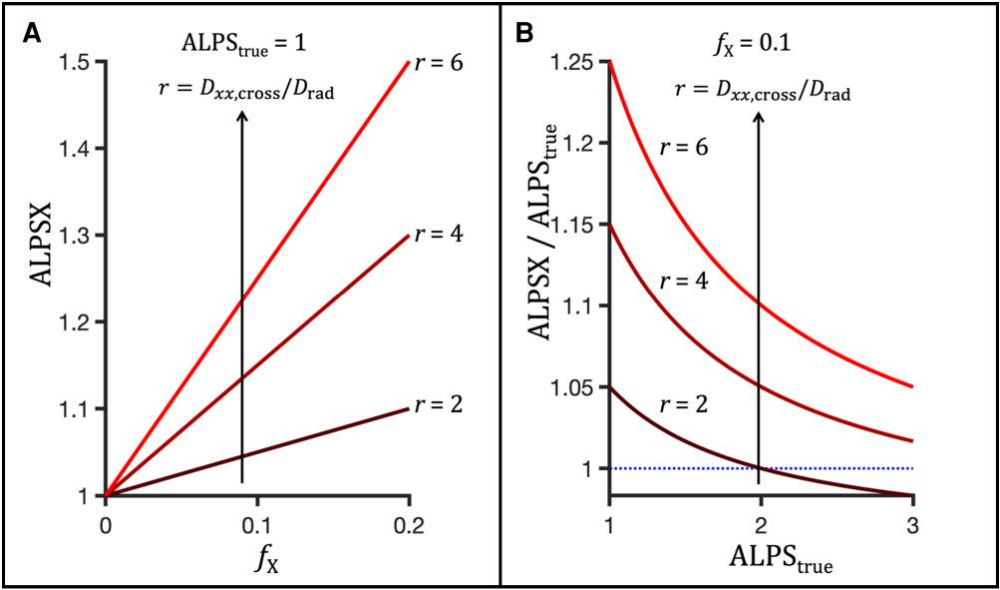
**ALPS bias due to fibres crossing along *x*-axis at a single site (association or projection fibres). A**. Apparent ALPS index (ALPSX) as measured in the presence of crossing fibres, as a function of the fraction of crossing fibres, $f_{X}$, and the anisotropy of crossing fibres ($r=D_{xx,\;\mathrm{cross}}/D_{\mathrm{rad}}$). **B**. Relative bias as a function of the true ALPS at a constant fraction of crossing fibres $f_{X}=0.1$. Note that the bias is positive and large for small true ALPS values. For less anisotropic crossing fibres and for large true ALPS values, the apparent ALPS index could in principle be lower than 1.

If fibres are crossing at both sites, so that $f_{\mathrm{Xp}}=f_{\mathrm{Xa}}=f_{X}$, the factor $\frac{\;f_{X}}{2}$ in Equation (5) becomes twice as large, i.e. $f_{X}$. According to Equation (5), for small $\mathrm{ALPS}<\frac{D_{xx,\mathrm{cross}}}{D_{\mathrm{rad}}}$ the bias is positive and proportional to the fraction and the diffusion anisotropy of the crossing fibres (see Fig. 2A). For larger ALPS indices, the bias tapers off and may in principle become negative (see Fig. 2B).

### Participants

Participants were recruited from the Parkinsonian symptoms’ cohort (*n* = 350) included in the Swedish BioFINDER-1 study (https://biofinder.se) by the Neurology Clinic at Skåne University Hospital, Sweden, as previously described.^31^ In this cross-sectional study, we included individuals diagnosed with PD or PSP as well as healthy controls, who had undergone at least one MRI that included DTI. When multiple MRI scans were available, the one closest to the participant’s enrolment date was selected. We included all available healthy controls (*n* = 41) and all available PSP patients (*n* = 17). The PD group (*n* = 60) was age-matched to the healthy control group. Patients with PD diagnosed with dementia at baseline or who developed PD dementia during the study were excluded. The diagnosis was made by neurologists trained in movement disorder diagnostics according to the National Institute of Neurological Disorders and Stroke criteria.^32,33^

### Diffusion-weighted MRI acquisition and processing

Diffusion-weighted imaging for subsequent DTI analysis was performed using a 3T Siemens Magnetom Skyra scanner with a 20-channel head coil. Data were acquired using a single-shot EPI (TE/TR 70/7500 ms) sequence with diffusion encoding in 30 directions using a *b*-value of 1000 s/mm^2^, iPAT factor of 2 and a voxel size of 2 × 2 × 2 mm^3^. Acquisition time was 4 min 15 s.

The diffusion data were preprocessed using in-house developed software in MATLAB R2022a (The MathWorks, Inc., Natick, MA, USA), available at https://github.com/markus-nilsson/md-dmri,^34^ in combination with other methods. Preprocessing steps included denoising,^35^ subject motion correction and eddy current correction,^36^ and generation of diffusion-encoded colour maps weighted by fractional anisotropy (FA). To account for the potential influence of crossing fibres, we generated a modified FA map, which caused regions with a strong presence of crossing fibres to become dark. Specifically, we reduced the brightness of the diffusion-encoded colour map to zero in voxels where the ratio of the middle to the lowest eigenvalue of the diffusion tensor exceeded 1.8. This empirically derived threshold was chosen to darken only voxels known to contain crossing fibres, based on previous literature.^37^ Simulation results of two diffusion tensors with variable crossing angles demonstrated that this index tends to zero when the angle between crossing fibres exceeds 50°. Notably, analyses of tools such as BEDPOSTX from FSL indicate that this is a typical threshold for detecting crossing fibres, even with more advanced methods than the one used here. The approach generated two colour-encoded diffusion maps for each patient: one conventional map and another adjusted to appear dark in regions with a strong presence of crossing fibres. These maps were subsequently used for ROI placement.

### ROI placement and calculation of DTI-ALPS index

**Figure 3:**
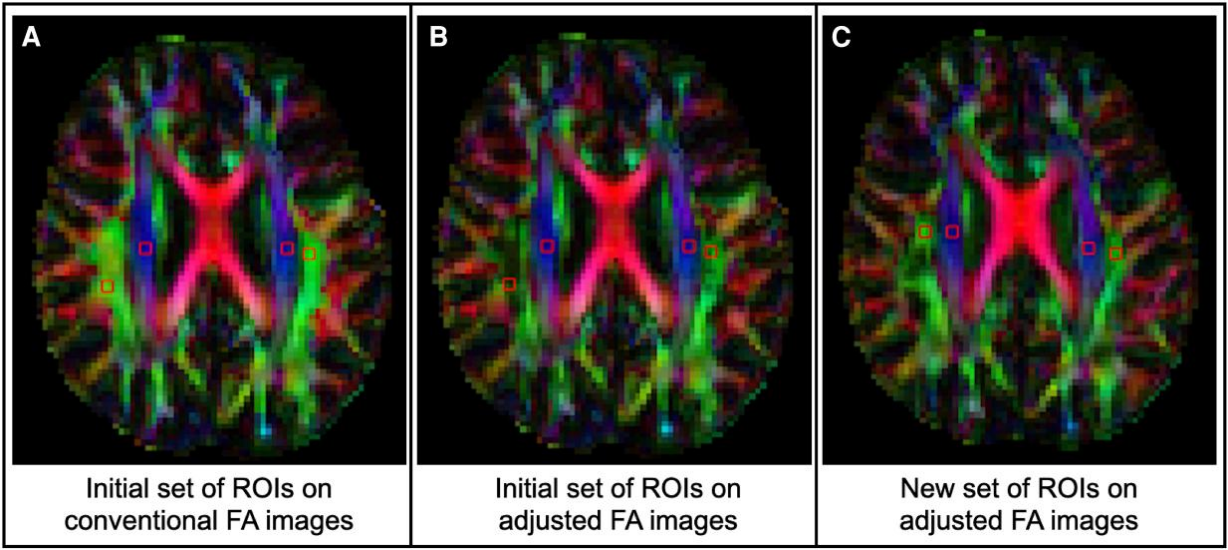
**An example of ROIs annotation on conventional and adjusted FA maps. A**. The ROIs were initially placed on conventional FA maps. **B**. After adjusting for crossing fibres by darkening the colour in areas with high level of crossing fibres, it becomes apparent that the initial set of ROIs includes voxels with high amount of crossing fibres. **C**. To avoid areas with crossing fibres, a new set of ROIs was annotated on the same participant based on the adjusted FA map.

Two regions of interest (ROI) were placed manually in each hemisphere to represent the projection and association fibres, according to the method described in the original ALPS paper.^6^ These ROIs were applied to both conventional and adjusted FA maps, blinded to the participants’ diagnoses and clinical assessment. We used square ROIs of 2 × 2 voxels, positioned at the level of the lateral ventricle body, on the slice displaying the clearest distinction of fibre types (Fig. 3). The ROI placement was carried out by AW, a final year medical student, under the close supervision of C.G., a neuroradiologist with 12 years of experience. Each case was discussed individually to ensure accurate ROI placement. An additional quality control was conducted by C.G. and M.N., an MRI physicist with 17 years of experience, to further validate the accuracy of the ROI placement. Furthermore, FLAIR images were assessed for white matter hyperintensities. All participants were classified as Fazekas grade 0 or 1, indicating the absence of severe hyperintensities. Additionally, no confluent hyperintensities were observed near the lateral ventricles, where the ROIs were placed.

In-house developed MATLAB R2022a scripts were used to compute the diffusivities (Dx, Dy, Dz) for each ROI and each hemisphere separately. Thus, for each participant, two ALPS indices were calculated: (i) a conventional ALPS index for each hemisphere, based on ROI placement on conventional FA maps, and (ii) an adjusted ALPS index, based on ROI placement on FA maps adjusted for crossing fibres.

### Statistical analysis

Statistical analysis was performed using IBM SPSS Statistics v.29.0 (IBM Corp, Armonk, NY, USA). To examine sex and age differences among diagnostic groups, we used the χ^2^ test and Kruskal–Wallis ANOVA, respectively. To examine differences in UPDRS and Hoehn and Yahr scores between PD and PSP patients, we used the Mann–Whitney U-test. DTI-ALPS indices across diagnostic groups were evaluated using MANCOVA, incorporating Bonferroni correction for pairwise comparisons. In this analysis, conventional ALPS and adjusted ALPS served as dependent variables, with diagnosis as the fixed factor and age as a covariate. Within each diagnostic group, the Wilcoxon signed-rank test was employed to compare conventional and adjusted DTI-ALPS indices. A similar approach was employed to test for group-wise differences in FA and mean diffusivity (MD) where MANCOVA with age and sex as covariates was used to detect potential differences among groups, whereas Wilcoxon signed-rank test was employed to compare those measures between conventional and adjusted ROIs. Furthermore, the correlation between the ALPS index and the third part of the UPDRS score for participants from the PD and PSP groups was separately assessed using Spearman’s rho. Significance level was set at *P* < 0.05.

## Results

Upon completing the sampling process, 118 patients were enrolled in the study, divided into three groups: healthy controls (*n* = 41), PD (*n* = 60) and PSP (*n* = 17). The demographics for each group are detailed in Table 2. There was no significant difference in sex distribution across the groups. However, a significant difference in age distribution was observed (*P* = 0.003), with pairwise comparisons revealing that the PSP group was significantly older than both the PD group (*P* < 0.001) and the healthy controls’ group (*P* = 0.007). No significant age difference was found between the PD and healthy controls’ groups (*P* = 0.491), as these had been matched with respect to age. PSP patients had significantly higher UPDRS score (*P* < 0.001) and Hoehn and Yahr score (*P* < 0.001) compared with patients with PD. There were no differences in terms of disease duration between PD and PSP patients. However, PD patients were in early stages of the disease, as indicated by the UPDRS and Hoehn and Yahr scores, whereas PSP patients were in later stages of the disease.

**Table 2:** Demographics

	PD	PSP	HC
*n*	60	17	41
Age (y)	63.3 ± 1.5	70.9 ± 1.5^a^	64.5 ± 8.4
Sex (male/female)	33/27	9/8	15/26
Disease duration (y)	5 ± 3.8	4.1 ± 2	n.a.
UPDRS, part 3	10.7 ± 8.3	37.8 ± 16.3^b^	n.a.
Hoehn and Yahr	1.6 ± 0.7	3.6 ± 1.3^b^	n.a.

Results are presented in the form of mean value ± standard deviation.

PD, Parkinson’s disease; PSP, progressive supranuclear palsy; HC, healthy controls; *n*, total number of participants; y, years; UPDRS, Unified Parkinson’s Disease Rating Scale; n.a., not applicable.

^a^PSP patients were significantly older than both the PD group (*P* < 0.001) and the HC group (*P* = 0.007).

^b^PSP patients had significantly higher UPDRS and Hoehn and Yahr scores compared with PD patients (*P* < 0.001).

When comparing all groups, there was a statistically significant difference in all ALPS indices, both conventional and adjusted, among the diagnostic groups: *F*(8, 220) = 2.998, *P* = 0.003, with Wilks’ Λ = 0.813 (adjusting for age and sex). For the conventional ALPS index, pairwise comparisons showed that PSP patients had a significantly lower ALPS index compared with both healthy controls (right hemisphere: *P* = 0.009; left hemisphere: *P* < 0.001) and PD patients (right hemisphere: *P* = 0.024; left hemisphere: *P* < 0.001). No significant differences were found between healthy controls and PD patients. For the adjusted ALPS index, PSP patients again showed significantly lower ALPS index compared with healthy controls (right hemisphere: *P* = 0.044; left hemisphere: *P* = 0.029) and PD patients (*P* = 0.003 for the left hemisphere only). Similarly, when comparing all groups, significant group differences were found in FA and MD: *F*(32, 196) = 1.883, *P* = 0.005, Wilks’ Λ=0.585 (adjusting for age and sex). Table 3 presents the ALPS indices, FA and MD for each diagnostic group, both in conventional and adjusted ROIs, with annotations for significant differences after pairwise comparisons.

**Table 3:** ALPS indices, along with FA and MD of projection and association fibres for each diagnostic group, before (conventional) and after (adjusted) avoiding areas with a high incidence of crossing fibres

	PD	PSP	HC
Right hemisphere			
Conventional ALPS index	1.51 ± 0.23	1.31 ± 0.20^a,b^	1.55 ± 0.21
Adjusted ALPS index	1.41 ± 0.19	1.26 ± 0.13^a^	1.43 ± 0.16
Conventional FA, association fibres	0.61 ± 0.07	0.58 ± 0.08^a^	0.62 ± 0.06
Adjusted FA, association fibres	0.61 ± 0.08	0.58 ± 0.09	0.62 ± 0.07
Conventional FA, projection fibres	0.48 ± 0.05	0.47 ± 0.08	0.46 ± 0.05
Adjusted FA, projection fibres	0.47 ± 0.06	0.48 ± 0.09	0.46 ± 0.06
Conventional MD, association fibres	0.71 ± 0.05	0.75 ± 0.11	0.70 ± 0.05
Adjusted MD, association fibres	0.71 ± 0.07	0.75 ± 0.10	0.71 ± 0.07
Conventional MD, projection fibres	0.70 ± 0.06	0.77 ± 0.09^a,b^	0.70 ± 0.06
Adjusted MD, projection fibres	0.71 ± 0.05	0.75 ± 0.09	0.71 ± 0.06
Left hemisphere			
Conventional ALPS index	1.54 ± 0.20	1.27 ± 0.23^a,b^	1.57 ± 0.18
Adjusted ALPS index	1.45 ± 0.19	1.25 ± 0.23^a,b^	1.43 ± 0.14
Conventional FA, association fibres	0.6 ± 0.06	0.59 ± 0.05	0.62 ± 0.06
Adjusted FA, association fibres	0.61 ± 0.06	0.58 ± 0.06	0.61 ± 0.07
Conventional FA, projection fibres	0.46 ± 0.05	0.48 ± 0.09	0.48 ± 0.06
Adjusted FA, projection fibres	0.46 ± 0.06	0.49 ± 0.09	0.48 ± 0.06
Conventional MD, association fibres	0.71 ± 0.05	0.73 ± 0.05	0.70 ± 0.05
Adjusted MD, association fibres	0.73 ± 0.06	0.74 ± 0.07	0.71 ± 0.06
Conventional MD, projection fibres	0.73 ± 0.04	0.77 ± 0.04^a,b^	0.70 ± 0.05
Adjusted MD, projection fibres	0.73 ± 0.05	0.78 ± 0.05^a,b^	0.72 ± 0.04

Results are presented in the form of mean value ± standard deviation.

PD, Parkinson’s disease; PSP, progressive supranuclear palsy; HC, healthy controls; ALPS, along the perivascular space.

^a^Statistically significant differences between PSP and HC (*P* < 0.05).

^b^Statistically significant differences between PSP and PD (*P* < 0.05).

**Figure 4:**
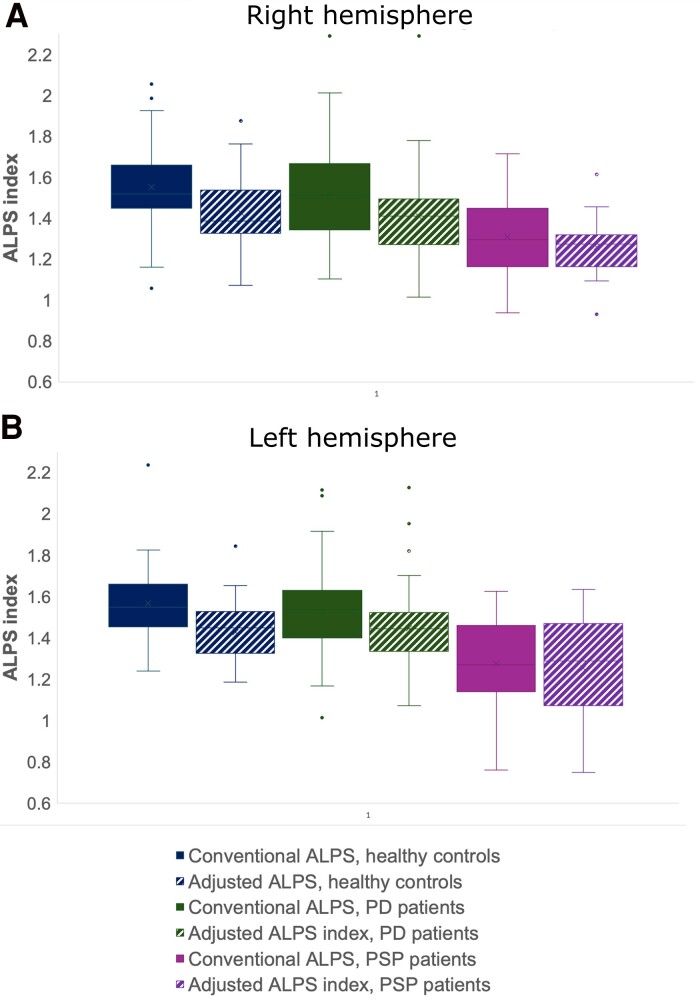
**Box and whisker plots of conventional and adjusted ALPS indices for each diagnostic group.** Adjusted ALPS index was significantly lower than conventional ALPS index in both healthy controls (*n* = 41) and patients with PD (*n* = 60) in both hemispheres. In PSP (*n* = 17), adjusted ALPS index was lower only in the right hemisphere. Statistical test: MANCOVA with conventional ALPS and adjusted ALPS as dependent variables, diagnosis as the fixed factor, age and sex as covariates and Bonferroni correction for pairwise comparisons. *: *P* < 0.05.

The adjusted ALPS index was significantly lower for healthy controls and Parkinson’s patients compared with conventional ALPS (Fig. 4): healthy controls (right hemisphere: *P* < 0.001; left hemisphere: *P* < 0.001) and PD (right hemisphere: *P* < 0.001; left hemisphere: *P* < 0.001). For PSP patients, the adjusted ALPS index was significantly lower only in the right hemisphere (*P* = 0.047). After adjusting the ROIs for crossing fibres, FA was slightly lower in left association fibres in healthy controls (*P* = 0.009), while MD was slightly higher in right projection fibres (*P* = 0.036).

Patients with PSP demonstrated moderate, positive correlation between the third part of the UPDRS score and the ALPS index only in the left hemisphere (conventional ALPS: *ρ* = 0.492, *P* = 0.045; adjusted ALPS: *ρ* = 0.591, *P* = 0.013). There was no significant correlation between the UPDRS score and any of the ALPS indices for the patients with PD.

## Discussion

In this study, we examined the influence of crossing fibres on the ALPS index. Based on the reasoning expressed in Equation 5, we hypothesized that excluding areas with high levels of crossing fibres from the measurement would decrease the ALPS index. This hypothesis was confirmed by our results, showing that avoiding areas with a high incidence of crossing fibres significantly reduces the ALPS index, across all diagnostic groups. Specifically, in comparisons of the ALPS index among healthy controls, PD patients and PSP patients, we found that the differences in the ALPS index were driven by PSP patients, while ALPS index in PD patients did not differ significantly from that of healthy controls. By showing that crossing fibres introduce a bias in the ALPS index, our results suggest that a low ALPS index when using conventional FA maps may simply reflect fewer crossing fibres, rather than impaired glymphatic function as initially thought.

The ALPS index is significantly affected by the degree to which crossing fibres are present in the ROIs used to compute the ALPS index. To our knowledge, this is the first study to demonstrate this. In the paper presenting the ALPS method, the authors emphasized the importance of correct ROI placement. To maintain reproducibility, they recommended to uniformly place ROIs in the projection and association fibres at the level of the lateral ventricular body across subjects.^26^ However, this strategy cannot be used to avoid regions of crossing fibres, as the presence of crossing fibres cannot be reliably detected from direction-encoded diffusion maps weighted by the FA (Fig. 1). Our results showed that avoiding regions with crossing fibres leads not only to a lower ALPS index but also to lower standard deviation within each diagnostic group, as given in Table 3. Therefore, to enhance the reproducibility of the ALPS index, it is essential to use a map that displays information on fibre crossing when placing ROIs.

Previous studies with DTI-ALPS show high variance, indicating a lack of consensus in this field (Table 1). Although Table 1 is not an exhaustive overview of ALPS studies in PD—some studies did not report ALPS indices quantitatively but only graphically and were therefore excluded^19,38^—one notable observation is the large variability in the effect size of the ALPS index, ranging from 0.19 to 0.9. Additionally, there is considerable variation in acquisition parameters, demonstrated by differences in repetition and echo times, and ROI placement, which is bilateral in some studies and only in the left hemisphere in others. The absence of a standardized approach to applying the DTI-APS method likely contributes to the variability in reported ALPS indices. Our study further explores this diversity, highlighting that the degree to which crossing fibres are present in the ROIs represent another critical factor that must be considered, and they may partly explain the variability observed in the effect sizes of previous studies.

Contrary to most previous literature, our study did not identify any differences in the ALPS index between patients with PD and healthy controls, regardless of the ROI placement strategy. A plausible explanation for this result may be attributed to differences between cohorts. Unlike the prior studies summarized in Table 1, our participants appear to be in an earlier stage of the disease, indicated by lower UPDRS scores. Notably, most previous studies do not report disease duration. However, previous studies have shown that the ALPS index is further reduced in patients with a Hoehn and Yahr stage >2^20^ and exhibits a negative correlation with both age at the time of the exam and age at disease onset.^18^ Similarly, one study that included two PD subgroups—one early and one late—reported differences only between late-stage patients with PD and healthy controls, with no significant differences observed between early-stage PD and healthy controls or between the early and late PD groups.^21^ Therefore, our results, along with earlier studies, suggest that ALPS index may not be a sensitive measure in the early stages of PD.

Conversely, it appears as disease severity has the largest impact on the ALPS index, which is further underscored by the consistently lower ALPS index observed in patients with PSP. Although both the PSP and PD patients had similar disease durations, PSP is marked by more rapid disease progression,^39^ as evidenced by the higher UPDRS score and Hoehn and Yahr stage. In PSP, neurodegeneration has been shown to affect even complex white matter tracts of crossing nature, such as the dentatorubrothalamic tracts.^40^ Furthermore, a recent study has demonstrated the presence of radial asymmetry within white matter tracts, which declines with age and neurodegeneration and mirrors changes in the ALPS index.^41^ These studies in combination with our findings suggest that a lower ALPS index might reflect broader effects of neurodegeneration rather than being solely indicative of specific glymphatic impairment.

Apart from crossing fibres, other factors can also influence the ALPS index. For instance, previous research has shown that the ALPS index decreases with age, even in individuals without neurological diseases.^42^ Despite adjusting for age in our statistical analysis, the potential influence of age could not be entirely ruled out, given that PSP patients were significantly older than the other subgroups. Our study further explores potential pitfalls of the ALPS methodology, indicating that ALPS is very sensitive to the amount and anisotropy of crossing fibres, which can inflate the ALPS index. This suggests that a lower conventional ALPS index might simply represent fewer crossing fibres within the ROI. For instance, our PD subgroup showed a 7–8% decrease in the adjusted ALPS index compared with the conventional ALPS index, a difference similar to that observed between healthy controls and PD patients in previous studies.^16,17,20,21,23^

The ALPS methodology has recently come under scrutiny.^43^ An editorial pointed out that the ALPS method focuses on the deep white matter, whereas glymphatic clearance predominantly occurs beneath the cortical surface, where the vascular network and its perivascular spaces are much denser.^27^ Even more recently, Taoka and his team have nuanced their claims about the ALPS index, stating that ‘*higher ALPS-index indicates predominant Brownian motion of water molecules in the radial direction at the lateral ventricular body level, no more and no less*’.^7^ Despite these critiques, the ALPS method remains particularly user-friendly and facilitates retrospective studies across various cohorts, leveraging extensive data collected over many years of DTI use. Our study does not seek to completely discard the ALPS method but instead aims to highlight the need for validation and consensus.

A limitation of our study was the relatively small size of our PSP cohort. Given the rarity of PSP as a diagnosis, expanding this group was not feasible. Consequently, we could not age-match these participants with other groups, leading to significant age differences. However, we adjusted for age in our comparisons of the ALPS index among groups, acknowledging the well-documented inverse correlation between ALPS index and age.^42^ Despite this, the robustness of our results is supported by the selection of our patients from a longitudinal cohort, which included a comprehensive and multimodal examination protocol. As a result, both patient groups and healthy controls are uniform; for instance, none of our PD patients has developed dementia, and none of the healthy controls has developed any neurodegenerative disorder over the course of 10 years. Another potential source of bias was the manual placement of ROIs, which is, however, considered standard practice in studies of this nature. To mitigate any bias related to the diagnosis, ROI placement was, however, performed without knowledge of the participants’ diagnoses. Furthermore, we acknowledge that the lack of susceptibility-induced geometric distortion correction in our MRI protocol is a limitation, although it is unlikely to have significantly affected our results, as the ROI annotation was performed directly on the diffusion-weighted images. Finally, future studies could incorporate tractography reconstructions to better assess the effect of crossing fibres on the ALPS index, which was not performed in our study.^44^ Additionally, more advanced diffusion methods that go beyond DTI may offer improved resolution of complex intravoxel fibre structures and enhance early differentiation between PSP and PD.^45^

## Conclusion

Our study highlights the role of crossing fibres as a pitfall in the ALPS method, which could potentially explain some of the variation observed in the existing literature. Our findings support recent criticism of the DTI-ALPS approach and underscore the need for further validation of this method. We advocate for its cautious use, ideally in conjunction with other proxies of glymphatic function, to ensure more accurate and reliable assessments. As glymphatic imaging advances, a variety of innovative techniques have emerged, ranging from the use of intrathecal and intravenous contrast agents to the automated segmentation of perivascular and peri-sinus structures.^46-49^ Continued refinement and validation of these methods will be essential for the field’s progress.

## Data Availability

Data supporting the findings of this study are available upon reasonable request to the corresponding author.
